# Technology transfer and scale down model development strategy for biotherapeutics produced in mammalian cells

**DOI:** 10.1186/1753-6561-7-S6-P86

**Published:** 2013-12-04

**Authors:** Nadine Kochanowski, Laetitia Malphettes

**Affiliations:** 1 Cell Culture Process Sciences Group, Biotech Sciences, UCB Pharma S.A., Braine L'Alleud, 1420, Belgium

## Background

The goal of manufacturing process development for drug substance and drug product is to establish a commercial process capable of consistently producing drug substance/drug product of the intended quality. Based on regulatory requirements, the manufacturing process has to be characterized prior to process validation. Since performing the characterization study at the manufacturing scale is not practically feasible, development of a scale down model that represents the performance of the commercial process is essential to achieve reliable process characterization. The developed scale down model could also be applied for cell line selection, process and medium development, raw material evaluation, limit of cell age studies, process parameter excursions, etc. Process development and commercial production should not be on the critical path to market despite the compressed time-to-market expectations. That is why Technology Transfer (TT) is a vulnerable time for companies. According to World Health Organization, Transfer of technology is defined as "a logical procedure that controls the transfer of any process together with its documentation and professional expertise between development and manufacture or between manufacture sites". In the pharmaceutical industry, Technology Transfer refers to the processes that are needed for successful progress from drug discovery to product development to clinical trials to full-scale commercialization or it is the process by which a developer of technology makes its technology available to commercial partner that will exploit the technology. This article describes the strategies and activities required to develop a scale down model. It also sketches a Technology Transfer approach for bioprocesses by focusing on the upstream part of a cell culture based process.

## Results

### Scale down model development strategy

"Small-scale models can be developed and used to support process development studies. The development of a model should account for scale effects and be representative of the proposed commercial process. A scientifically justified model can enable a prediction of quality, and can be used to support the extrapolation of operating conditions across multiple scales and equipment [2]. The key elements for designing a scale down model are inputs (raw materials and components, cell source, environmental conditions) and outputs (performance and product quality metrics, sample handling/storage, analytical methods). A scale down model can be equivalent for some outputs but not for all and still be a representative model. It should reproduce at small scale the effect/impact seen at large scale. The acceptability of an observed offset has to be statistically evaluated and scientifically understood.

### Technology Transfer strategy

**Figure 1:**
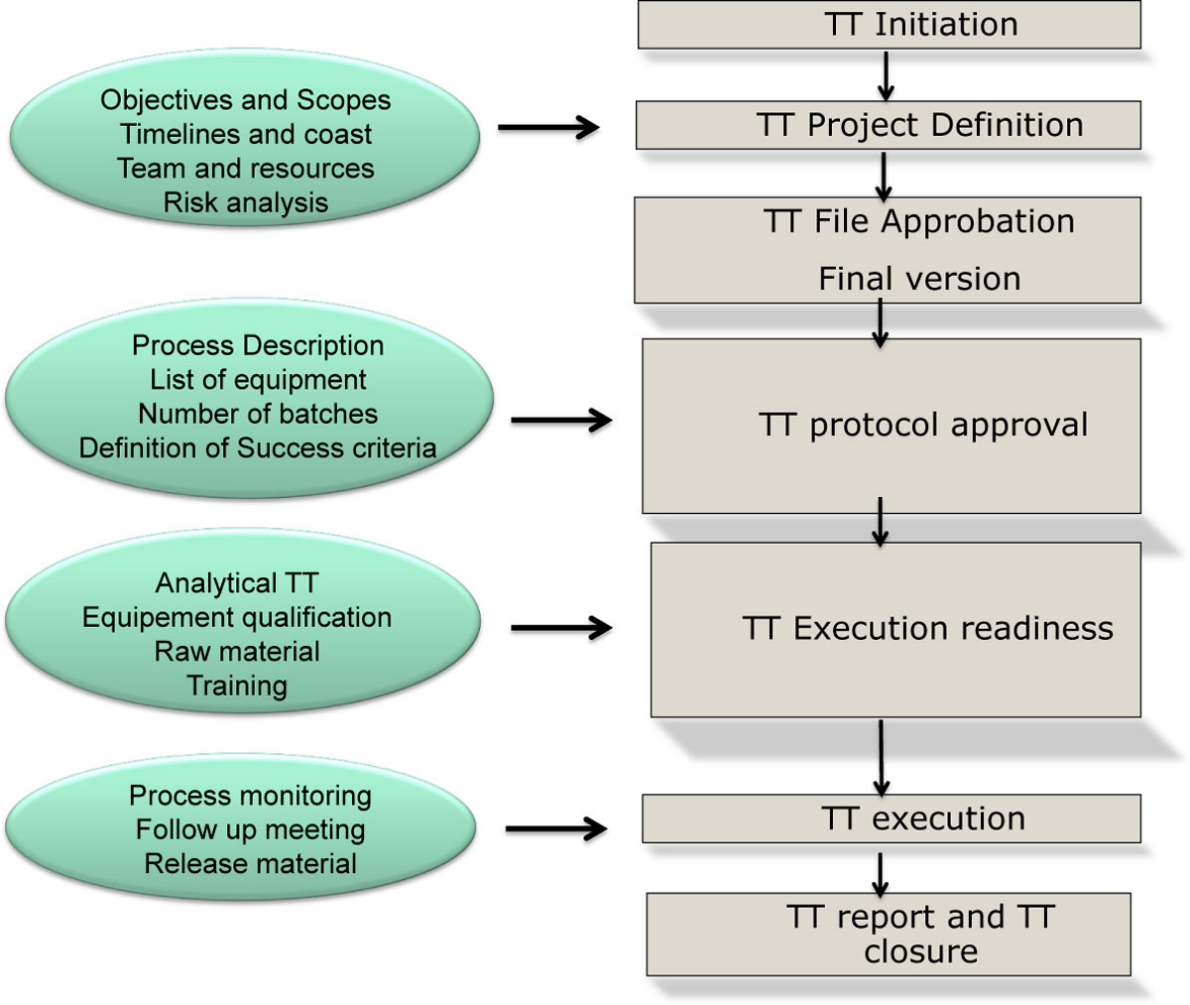
**Technology Transfer (TT) process flow chart**.

"The goal of Technology Transfer activities is to transfer product and process knowledge from development to market, and within or between manufacturing sites to support product commercialization. This knowledge forms the basis for the manufacturing process,control strategy, process validation approach and ongoing continual improvement [1]. A dedicated Technology Transfer team has to be set up to facilitate and execute the process including experts in different fields (production, QA, QC, RA, MSAT, etc.). The whole Technology Transfer has to be coordinated by the technology transfer/project leader. Organization for Technology Transfer should be established and composed of both party members from both sites, roles and scope of responsibilities of each party should be clarified, and adequate communication and feedback of information should be ensured. Figure 1 describes the main steps of the Technology Transfer. Technology Transfer can be considered successful if the Receiving Unit can routinely reproduce the transferred product, process, or method against a predefined set of specifications as agreed with the Sending Unit. The success of a Technology Transfer project will be largely dependent on the skill and performance of individuals assigned to the project from the Sending Unit and the Receiving Unit. The roles and responsibilities of the sending unit and the receiving unit have to be clearly defined. The documentation is a key element of Technology Transfer: it ensures consistent and controlled procedures for Technology Transfer and to run the process. Clear documentation should provide assurance of process and product knowledge (Table 1).

**Table 1:** Technology transfer documentation

Document	Content
Bill of materials	List of all components and their step of use (Supplier, grade)

Research and Development reports	Historical data of pharmaceutical development of new drug substances and drug products at stage from early development to final application of approval - Quality profiles of manufacturing batches (including stability data) - Specifications and test methods of drug substances, intermediates, drug products, raw materials and components, and their rationale - Change histories of important processes and control parameters

Risk assessment	Process flow charts - Scale up - Equipment changes - Media and feed preparation

Process descriptions	Product information - Process step flow diagram - Cell culture steps description (cell line/ inoculum/expansion/production bioreactor - Media and feed preparation - Harvest description - Raw materials/equipment)

Technology transfer file	Introduction - Manufacturing process description, process parameters - Equipment - Raw materials - Analyses - Safety, environment - Stability (conditions, results) - Packaging (cold chain requirements, etc...) - Cleaning - Shipment characteristics and proper validation if needed - Historical data available

Technology transfer protocol	Technology transfer description - Scope - Objective - Responsibilities - Process Description - Equipment list (receiving unit) - Raw material list - Reference of Master batch record/number of repetitions and status of batches/acceptance criteria/relevant specifications/description of coaching

Manufacturing and testing description of the process	Product information - Process step flow diagram - Cell culture steps description (cell line/inoculum/cell expansion/production bioreactor) - Media and feed preparation - Harvest description (holding time/storage conditions) - Raw materials - Equipments

Routine and non-sampling plans	List of all the samplings that should be taken and kept in addition to the in-process control samples listed in the manufacturing description

Data recording list	Online and offline data to be monitored and recorded during the process

Deviation inventory	Description in details of the deviations and reporting of the impact on the product titer and quality

Technology transfer report	Technology transfer description - Objective - Scope - List of deviations and discussion - Process results and comparison to acceptance criteria -, Conclusions

## Conclusions

A scale down models is a tool for developing and characterizing the process and should be designed and demonstrated as appropriate representations of the manufacturing process. The transfer of technology from R&D to the commercial production site is a critical process in the development and launch of a biotherapeutical product. The three primary considerations to be addressed during an effective technology transfer are the project plan, the people involved and the process.
